# Epidemiology, Diagnosis, and Control of Canine Infectious Cyclic Thrombocytopenia and Granulocytic Anaplasmosis: Emerging Diseases of Veterinary and Public Health Significance

**DOI:** 10.3390/vetsci8120312

**Published:** 2021-12-08

**Authors:** Farhan Ahmad Atif, Saba Mehnaz, Muhammad Fiaz Qamar, Taleeha Roheen, Muhammad Sohail Sajid, Syed Ehtisham-ul-Haque, Muhammad Kashif, Mourad Ben Said

**Affiliations:** 1 Medicine Section, Department of Clinical Sciences, College of Veterinary and Animal Sciences, Jhang, Sub-Campus University of Veterinary and Animal Sciences, Lahore 54600, Pakistan; sabamehnaz2012@gmail.com (S.M.); muhammad.kashif@uvas.edu.pk (M.K.); 2 Department of Parasitology, Faculty of Veterinary Science, University of Agriculture, Faisalabad 38000, Pakistan; drsohailuaf@uaf.edu.pk; 3 Department of Pathobiology, College of Veterinary and Animal Sciences, Jhang, Sub-Campus University of Veterinary and Animal Sciences, Lahore 54600, Pakistan; fiaz.qamar@uvas.edu.pk (M.F.Q.); ehtishamsyed@uvas.edu.pk (S.E.-u.-H.); 4 Department of Chemistry (Biochemistry), University of Sargodha, Sargodha 40100, Pakistan; taleeharoheen@yahoo.com; 5 Higher Institute of Biotechnology of Sidi Thabet, University of Manouba, Manouba 2010, Tunisia; 6 Laboratory of Microbiology at the National School of Veterinary Medicine of Sidi Thabet, University of Manouba, Manouba 2010, Tunisia

**Keywords:** canine anaplasmosis, *Anaplasma platys*, *Anaplasma phagocytophilum*, diagnosis, epidemiology, control

## Abstract

This review highlights the diagnostic methods used, the control strategies adopted, and the global epidemiological status of canine cyclic thrombocytopenia and granulocytic anaplasmosis at the animal–human interface. Canine anaplasmosis is an important worldwide disease, mainly caused by *Anaplasma platys* and *A. phagocytophilum* with zoonotic implications. *A. platys* chiefly infects platelets in canids, while *A. phagocytophilum* is the most common zoonotic pathogen infecting neutrophils of various vertebrate hosts. Diagnosis is based on the identification of clinical signs, the recognition of intracellular inclusions observed by microscopic observation of stained blood smear, and/or methods detecting antibodies or nucleic acids, although DNA sequencing is usually required to confirm the pathogenic strain. Serological cross-reactivity is the main problem in serodiagnosis. Prevalence varies from area to area depending on tick exposure. Tetracyclines are significant drugs for human and animal anaplasmosis. No universal vaccine is yet available that protects against diverse geographic strains. The control of canine anaplasmosis therefore relies on the detection of vectors/reservoirs, control of tick vectors, and prevention of iatrogenic/mechanical transmission. The control strategies for human anaplasmosis include reducing high-risk tick contact activities (such as gardening and hiking), careful blood transfusion, by passing immunosuppression, recognizing, and control of reservoirs/vectors.

## 1. Introduction

Anaplasmosis is a vector-borne disease that affects animals and humans worldwide [1]. It is a virulent non-contagious disease caused by strictly intracellular Gram-negative bacteria. These pathogens parasitize circulating blood cells (erythrocytes, monocytes, granulocytes, and platelets) [2]. Ticks act as natural vectors for *Anaplasma* species and play a key role in the biological multiplication of these bacteria in salivary glands and guts [3]. The genus *Anaplasma* (*A.*) consists of several classified species that have a valid taxonomic standing, namely, *A. marginale*, *A. centrale*, *A. bovis*, *A. ovis*, *A. caudatum*, and *A. phagocytophilum* [1]. Infectious canine cyclic thrombocytopenia and granulocytic anaplasmosis are two zoonotic diseases caused by *A. platys* and *A. phagocytophilum*, respectively, mainly affecting dogs and wild canids [1,2]. *Anaplasma platys* was first detected in a dog from Florida and frequently infects platelets. The disease is characterized by fever, anorexia, weight loss, lethargy, petechiae, pale mucous membranes, nasal discharge, bilateral uveitis, epistaxis, and lymphadenomegaly [4,5]. However, *A. phagocytophilum* primarily infects canine granulocytes (especially neutrophils) of a wide range of domestic and wild vertebrate hosts, as well as humans [6]. The first case of human granulocytic anaplasmosis was observed in 1994 in tangential blood smears from six human patients of Wisconsin and Minnesota, states of the United States of America [7]. However, the first case of *A. platys* infection in humans was reported in 1992 [8]. After this, the organism was detected in a veterinarian and two family members of dog owners [9,10]. In 2017, there were 5762 cases of human anaplasmosis in the U.S., and now, global distribution of cases has been achieved [11]. In addition, transplacental transmission has also been reported for *A. phagocytophilum* [12].

Infection with *A. phagocytophilum* in dogs is known as canine granulocytic anaplasmosis. The host range of *A. phagocytophilum* includes ruminants, humans, carnivores, reptiles, birds, and rodents [13]. The increased prominence of human infections, alternative treatment options, availability of whole genome data, and alternative/promising preventive measures are all important contributions, and could perhaps be stressed earlier. Therefore, it is imperative to mention the updated global epidemiological status, diagnosis, and control of canine anaplasmosis at the animal–human interface.

## 2. History

In 1910, Sir Arnold Theiler discovered bacteria of the *Anaplasma* genus. He was the first to observe these “marginal spots” in the red blood cells of South African cattle, which he called *Anaplasma marginale* [14]. He later described *A. centrale* as subspecies of *A. marginale*, which seems to be less pathogenic and localized more frequently in the center of red blood cells than in the margins of erythrocytes [14].

In 1932, Gordon and his colleagues first noticed a disease in sheep in Scotland without identifying the causative agent in louping ill-affected districts transmitted by *Ixodes ricinus.* Further investigation revealed pathogens in the blood, spleen, and central nervous system. Later, the disease was diagnosed as tick fever, and the clinico-pathological aspects of the disease were studied in detail [14,15,16].

In 1949, Foggie placed this pathogen in the Rickettsial group, since the disease is transmitted by ticks, and named it *Ehrlichia phagocytophila ovis* [17]. The designation Ehrlichiae was chosen in honor of the German microbiologist Paul Ehrlich [18]. In 1969, Gribble discovered, in California (U.S.), a bacterium of the *Ehrlichia* genus causing a fatal disease in horses. He called the disease “equine granulocytic ehrlichiosis” in reference to the location of the morulae in the granulocytes [19,20]. The bacterium was subsequently described and named *Ehrlichia equi* by Lewis and his colleagues in 1975 [20]. In 1994, Chen and his colleagues in the U.S. diagnosed clinical case of ehrlichiosis in a human patient; they named it human granulocytic ehrlichiosis (HGE) [7,21].

## 3. Etiology

The word *Anaplasma* is derived from the Greek words *an* and *plasma*; the former means “without” and the latter means “molded.” *Anaplasma*, *Ehrlichia*, *Wolbachia*, and *Neorickettsia* belong to obligate intracellular bacteria that invade blood cells. *Anaplasma* is an obligate intracellular, Gram-negative alpha-proteobacteria that belongs to the Anaplasmataceae family, order Rickettsiales. Currently, *A. marginale*, *A. bovis, A. ovis, A. platys,* and *A.phagocytophilum* are important species of the genus *Anaplasma* (Table 1).

The sequencing of the whole genome of *A. phagocytophilum* was evaluated and shown to contain a 1.2–1.5 × 10^6^ bp circular chromosome [34]. Likewise, *A. platys* has a1.196 × 10^6^ bp genome size [35]. *Anaplasma platys* frequently infects dogs; however, it has also been reported in cats, camels, and humans. Nonetheless, *A. ovis* has also been described in humans [36]. *Anaplasma* spp. demonstrates some level of host specificity; this attribute is altered due to the detection of *Anaplasma* species in various hosts, which further complicates the pathology and epidemiology of the disease [36].

## 4. Taxonomical Position of *Anaplasma* Bacteria

### 4.1. Evolution of Taxonomy

The Anaplasmataceae family is included in the order Rickettsiales, obligate intracellular bacteria that exist in eukaryotic cells. Morphologically (Gram-negative bacteria) and epidemiologically, they have a particular tropism for blood cells, since all of them are mainly transmitted by ticks. This classification of Anaplasmataceae is based on the pathogenic characteristics of these bacteria, which are strictly intracellular [37]. Studies of Weisburg and Sumner and colleagues have revealed that, in reality, *Ehrlichia phagocytophila* and *Ehrlichia equi* are genetically similar to the etiologic agent of human granulocytic anaplasmosis [38,39]. Based on the sequencing and phylogenetic proximity of these bacterial operons/genes (*groESL*, *gltA*, *ankA,* and *16S ribosomal RNA*), Dumler and his collaborators made profound taxonomic changes, leading to the reorganization of most family members, genera, and species of Rickettsiales [40].

### 4.2. Current Classification

Carrade and his collaborators reorganized the classification on the basis of their nucleic acid sequences, known antigenic properties, ecology, geographical distribution, and their pathogenicity. They mainly used the *16S rRNA* gene and *groESL* operon, and divided this order into two families. One is Rickettsiaceae, which remains free in the cytoplasm, and the second is Anaplasmataceae, which is contained in a vacuole bound to the cytoplasmic membrane derived from the host cell [41]. The present classification is based on the phylogenetic analysis of the *16S rRNA* and *groESL* genes replacing and renaming *Ehrlichia* (*E*.) *bovis*, *E. platys*, and *E. phagocytophilum* as *A. bovis*, *A. platys*, and *A. phagocytophilum,* respectively [40].

## 5. Epidemiology

Anaplasmosis has a worldwide distribution, and is potentially endemic in forty-three countries of the world [42]. Although, the prevalence varies among area, species, breeds, due to the presence of different ticks, and diagnostic assays involved. *Anaplasma phagocytophilum* and *A. platys* have been identified on all continents. Sufficient scientific data are available for *A. phagocytophilum,* while less epidemiological and risk factor information is available for *A. platys*. Nevertheless, *A. phagocytophilum* can infect a wide range of wild/domestic animals and humans; however, *A. platys* typically infects dogs and rarely cats [13,43]. Animals recover from acute anaplasmosis, develop a lifelong persistent infection with low cyclic rickettsiemia, and act as a reservoir host for further spread [44]. *A. platys* has zoonotic potential and there are reports of human infection to a lesser extent [45].

### 5.1. Anaplasma platys

*Anaplasma platys* was first observed in a blood test of a dog in the United States in 1978. It has been detected on almost all continents with worldwide distribution [4,46,47,48,49,50,51]. *A. platys* widely infects dogs; however, it has also been shown in deer, cats, cattle, and humans [5]. In dogs, severe thrombocytopenia results in recrudescence after two weeks of incomplete recovery. Thrombocytopenia can occur as a result of direct damage to platelets and immune cells caused by immune-mediated mechanisms [4]. In dogs and cats, the serological and molecular prevalence rates range from 0.4% to 87.5% and from 0.6% to 6.6%, respectively, depending on the region, breed, and involved test (Table 2). Regarding the infected host, the overall prevalence and distribution of *A. platys* in domestic canids are shown in Table 2. Interestingly, the camel is an animal species that significantly harbors canine *A. platys* and various *A. platys*-like strains during natural infection [52,53,54]. It is necessary to determine the pathogenicity and the epidemiological role of camelids in the transmission of this *Anaplasma* species.

### 5.2. Anaplasma phagocytophilum

*Anaplasma phagocytophilum* is one of the most diverse pathogens infecting humans, and domestic and wild animals. This species is most widespread in northern Europe. Small mammals play a vital role in disease transmission. *A. phagocytophilum* is mainly transmitted by tick bites [55]. Disease outcome and response to treatment are complex in dogs, co-infected with *H. canis*, *B. vogeli*, and/or *Ehrlichia canis*. After the incidence of animal anaplasmosis in an area, the screening of human anaplasmosis should be considered. Sero-surveillance has shown a prevalence of 15–36% in humans with an annual incidence of approximately 58 cases per 100,000 individuals in the U.S. [18]. The rate of human infection increases with infected vectors. Indeed, there is a high rate of incidence of human granulocytic anaplasmosis (HGA) in the U.S. There were approximately 2782 cases of HGA recorded during the year 2013 [11].

Regarding HGA, patients’ clinical signs range from asymptomatic to severe clinical disease, and approximately40% of patients require hospitalization [18,56,57]. The mortality rate in the U.S. ranges from 7% to 10% [58,59,60]. The severity of infection depends on the phase of bacterial growth, the susceptibility of the host, and the pathogenic bacterial strain. A blood test of the infected host reveals that there is a decrease in the number of neutrophils and leukocytes, resulting in immunosuppression and a tendency of opportunistic infection [61]. Approximately30% of the patients required prompt hospitalization due to the development of life-threatening conditions, including severe sepsis, anaphylactic shock, and respiratory syndrome [57]. Death occurs mainly due to a combination of other health problems, including intravascular coagulation, kidney failure, enlarged heart, coma, and seizures.

**Table 2:** Detection of *A. platys* in domestic canid hosts from different countries *.

Domestic Canid	Countries (Region)	Prevalences (%)	Methods (Target Genes)	References
Dog	Thailand	13.9	PCR^a^ (*groEL*)	[62]
	Thailand	29.4	PCR^a^ (*16S rRNA*)	[63]
	Thailand	7.0	PCR^a^ (*16S rRNA*)/mHRM^b^	[64]
	West Indies (Grenada)	18.7	PCR^a^ (*16S rRNA*)	[65]
	West Indies (Grenada)	33.0	PCR^a^ (*16S rRNA*)/ELISA^c^	[66]
	West Indies (Grenada)	16.4	RT-PCR^d^ (*16S rRNA*)	[67]
	West Indies (Trinidad)	2.3	PCR^a^ (*16S rRNA*)/RBL^e^	[68]
	Pakistan	11.34	PCR^a^ (*16S rRNA*)	[69]
	Paraguay	10.67	PCR^a^ (*16S rRNA*)	[70]
	Colombia	20.2	RT-PCR^d^ (*16S rRNA*)	[71]
	Greece	Case report	Blood smear/ELISA^c^	[72]
	Indonesia	11.76	PCR^a^ (*groEL*)	[73]
	Cape Verde	7.7	PCR^a^ (*16S rRNA*)	[74]
	Italy	70.5	PCR^a^ (*groEL*)	[75]
	Italy (Putignano)	52.9	RT-PCR^c^ (*16S r RNA*)	[76]
	Italy (Teramo Kennel)	33.0	PCR^a^ (*16S rRNA*)/RLB^e^	[48]
	Croatia	Case report	RT-PCR^d^ (*groEL*)	[77]
	Australia	51.3	RT-PCR^d^ (*16S rRNA*)	[78]
	Australia	23.7	ELISA^c^	[78]
	Australia	32.0	PCR^a^ (*16S*/*18S rRNA*)	[49]
	Australia	3.8	Blood smear/ELISA^c^/PCR^a^	[79]
	Romania	Case report	PCR^a^ (*16S rRNA*)	[80]
	Dominican Republic	11	RT-PCR^d^ (*16S/18S rRNA*)	[81]
	Nicaragua	13	RT-PCR^d^(*16S/18S rRNA*)	[82]
	Caribbean	10.3	ELISA^c^	[83]
	Canada	1.8	ELISA^c^	[83]
	USA (South)	2.0	ELISA^c^	[83]
	USA (Mid Atlantic)	1.1	ELISA^c^	[83]
	USA (Northeast)	1.5	ELISA^c^	[83]
	USA (Midwest)	0.6	ELISA^c^	[83]
	USA (West)	1.0	ELISA^c^	[83]
	Mexico	31.0	PCR^a^ (*16S rRNA*)	[84]
	Brazil	7.19	PCR^a^ (*16S rRNA*)	[51]
	Turkey	0.5	RLB^d^	[85]
	Costa Rica	1	PCR^a^ (*16S rRNA, groEL*)	[86]
	Brazil	16.96	nPCR^f^ (*16S rRNA*)	[87]
	Brazil	19.4	PCR^a^ (*16S rRNA*)	[88]
	Brazil	14.07	nPCR^f^ (*16S rRNA*)/ELISA^c^	[89]
	Colombia	53.0	PCR^a^ (*16S rRNA*)/ELISA^c^	[90]
	Palestine	53.0	PCR^a^ (*16S rRNA*)	[91]
	China	62.1	RT-LAMP^g^/nPCR^f^ (*16S rRNA*)	[92]
	Caribbean	18.7	PCR^a^ (*16S rRNA*, *gltA, groEl)*	[65]
	Argentina	37.5	PCR^a^ (*16S rRNA*, *groESL*)	[93]
	Costa Rica	6.25	nPCR^f^ (*16S rRNA*)/ELISA^c^	[94]
	Myanmar	0.25	PCR^a^ (*16S rRNA*)	[95]
	Malawi	2.4	PCR^a^ (*16S rRNA*)	[96]
	Galápagos	6.9	PCR^a^ (*16S rRNA*)/ELISA^c^	[97]
	Saudi Arabia	57.1	RT-PCR^c^ (*16S rRNA*)	[98]
	Greek islands	18.0	PCR^a^ (*16S rRNA*)/IFAT^h^	[99]
	Malta	22.7	PCR^a^ (*16S rRNA*, *cox1*)	[100]
	Haiti	6.3	PCR^a^ (*16S/18S rRNA*)	[101]
	Cambodia	32.0	NGS^i^ based metabarcoding	[102]
	Uganda	18.9	RT-PCR^d^ (*16S rRNA*)/IFAT^h^	[103]
	Albania	3.3	PCR^a^ (*16S rRNA*)/ELISA^b^	[104]
	Nigeria	6.6	RT-PCR^d^ (*16S rRNA*)	[105]
	Qatar	1.6	PCR^a^ (*16S rRNA*)	[106]
	Texas	0.17	RT-PCR^d^ (*16S rRNA*)	[107]
	India	22.6	PCR^a^ (*16S rRNA*)	[108]
	Japan	32.0	PCR^a^ (*16S rRNA*)	[109]

^a^ Polymerase chain reaction; ^b^ multiplex high-resolution melting analysis; ^c^ enzyme-linked immunosorbent assay; ^d^ real-time polymerase chain reaction; ^e^ reverse line blot hybridization; ^f^ nested polymerization chain reaction; ^g^ real-time loop-mediated isothermal amplification; ^h^ indirect fluorescent antibody test; ^i^ next-generation sequencing based on metabarcoding. * Detection of *A. platys* from 1991 up to date.

The disease is more severe in elderly patients and immunocompromised children [58,59].

*Anaplasma phagocytophilum* is mainly transmitted by ixodid ticks of the genera *Ixodes*, *Dermacentor*, *Haemaphysalis*, and *Amblyomma* in Europe, the U.S., and Asia [24]. In ticks, transstadial transmission occurs [110,111], while other routes of transmission are less common, such as contact with infected blood and tissues [57,112]. Serological and molecular prevalence rates vary from 0.3% to 55.6% for dogs and 0.9% to 37.6% for cats depending upon the area, breed, and test used (Table 3). The infected host, the global prevalence of infection, and the distribution of *A. phagocytophilum* in domestic canid hosts are listed in Table 3.

## 6. Transmission

Ixodidae ticks act as biological vectors and play an essential role in the spread and propagation of *Anaplasma* during various stages of its life cycle [121]. Nonetheless, vertical transmission has also been reported for *A. platys* infection in bitches during early gestation (25–35 days) and intrauterine transmission for *A. phagocytophilum* as well [122,123,124]. Vertebrates are definitive hosts and also serve as reservoirs [125]. *Rhipicephalus* (*R.*) *sanguineus* and *I. ricinus* are the major vectors of *A. platys* and *A. phagocytophilum*, respectively [124,126].

## 7. Life Cycle

The life cycle of all *Anaplasma* species hasnot yet been completely studied. Most studies have been performed on *A. marginale* in cattle in association with *R. microplusticks*. The life cycle begins with the ingestion of *Anaplasma* by tick vectors during a blood meal [127]. *A. phagocytophilum* frequently infects granulocytes, causing leukopenia and thrombocytopenia. This changes the host’s immune system and positively regulates cellular cholesterol and several tick genes.

However, *A. platys* primarily infects platelets causing thrombocytopenia, and can also infect megakaryocytes and promegakaryocytes [127]. Transstadial, transovarial, and mechanical propagation, as well as several other host-related factors, make the conditions essential for the maintenance of *Anaplasma* in nature [128].

## 8. Clinical Findings

In dogs, *A. platys* causes canine cyclic thrombocytopenia with variable signs of fever, anorexia, weakness, anemia, lethargy, eye discharge, spot hemorrhage on the eye, oral mucosa and skin, respiratory distress, lymphadenomegaly, epistaxis, splenomegaly, and muzzle hyperkeratosis [46,129,130]. Thrombocytopenia may occur as a result of direct damage to platelets by the pathogen and immune-mediated systems [4]. Camels infected with *A. platys* generally remain asymptomatic, with some evidence of anorexia, dullness, progressive loss of physical condition, and stamina, as well as neutrophilia and eosinophilia [22]. Canine granulocytic anaplasmosis shows signs of high fever, vomiting, diarrhea, loss of appetite, lameness, polyuria, jaundice, epistaxis, lymphatic adenomegaly, and splenomegaly [131,132]. Cats show no specific clinical signs; however, signs of anorexia, fever, lethargy, and dryness with neutrophilia, lymphopenia, thrombocytopenia, and hyperglycemia can be observed [133,134,135].

In humans, *A. phagocytophilum* causes human granulocytic anaplasmosis. Patients present with flu-like symptoms ranging from asymptomatic to severe clinical illnesses. High fever, severe headache, stiff neck, myodynia, restlessness, cough, nausea, and vomiting are important clinical signs, and even diarrhea, joint pain, and neurological signs [136,137]. During illness, certain threatening conditions can develop in patients due to opportunistic pathogens. Often, laboratory tests are needed to maintain the diagnosis. Approximately 30% of the patients require hospitalization due to anaphylactic shock, severe sepsis, and respiratory syndrome [57]. The disease mortality rate is 7–10% in the United States [58,60]. Death occurs mainly due to the combination of other health problems related to intravascular coagulation, kidney failure, enlarged heart, coma, and seizures. The disease is more harmful in elderly immunocompromised patients [112].

## 9. Diagnosis

Diverse conventional, serological, and molecular methods have been validated for causative agent identification and disease diagnosis.

### 9.1. Direct Detection

**Figure 1:**
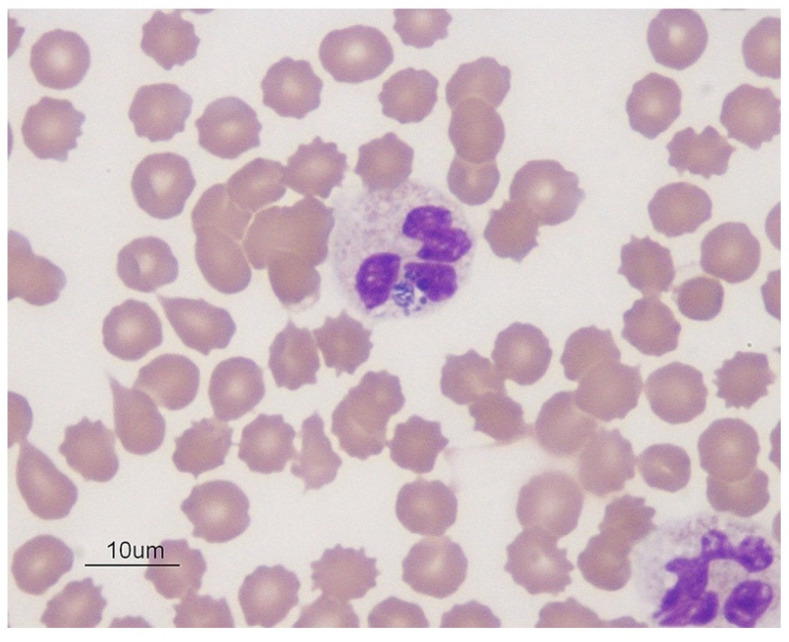
Blue color *Anaplasma phagocytophilum* in the cytoplasm of neutrophils in dog blood; Wright’s stain, 1000× (source: https://eclinpath.com; accessed on 11 August 2021; Cornell University College of Veterinary Medicine).

**Figure 2:**
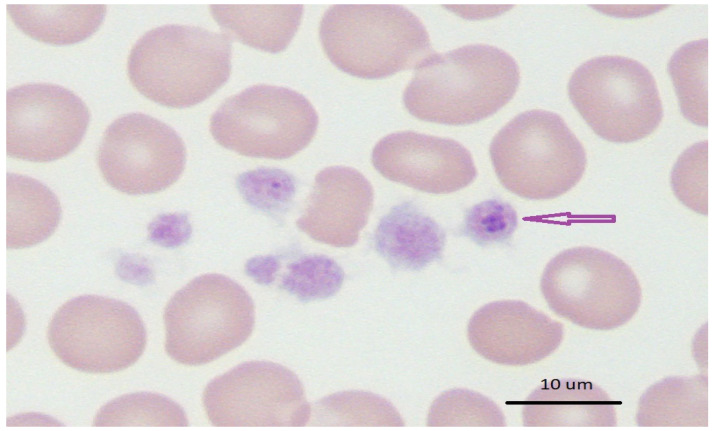
Blue–purple inclusions of *Anaplasma platys* in dog blood with thrombocytopenia; Wright’s stain 1000× (source: https://eclinpath.com; accessed on 11 August 2021; Cornell University College of Veterinary Medicine).

**Figure 3:**
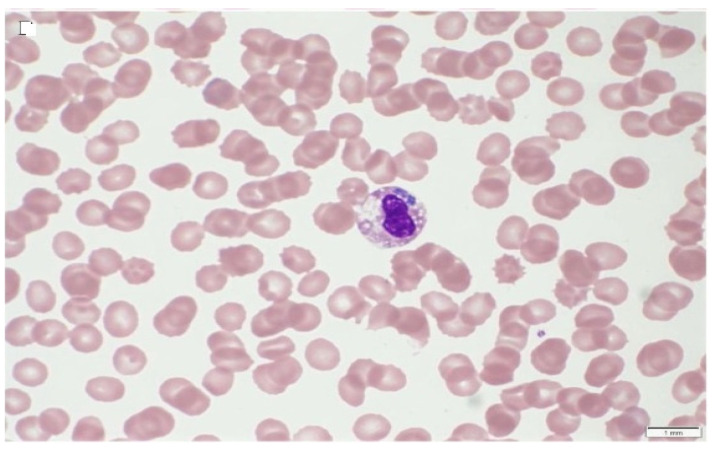
Human *A. phagocytophilum* infection, indicating morulae in infected neutrophils [142].

Conventional light microscopy of freshly prepared stained blood smears (Giemsa, Diff-Quik) taken from a vein are used for diagnosis in the acute phase of the disease (Figure 1, Figure 2 and Figure 3). *A. phagocytophilum* leads to the development of “morulae,” which are a combination of mulberry-type colonies formed in the neutrophils and eosinophils of infected organisms [61].

Typically, *Anaplasma* morulae resembledark blue to purple inclusion bodies. Conversely, refrigerated samples mixed with anticoagulants can be processed within 24–48 h. This is a quick, inexpensive, and best way to directly visualize bacteria before the start of antibacterial treatment. However, this method is less sensitive to lower bacteremia during persistent infection with monocytopenia, neutropenia, thrombocytopenia, and anemia [138,139]. The sample collection time is critical for the direct identification of bacteria involving microscopy, *in vitro* culture, and nucleic acid detection in order to detect sufficient number of organisms in the circulating blood [140]. Leukocyte smears would be a good option for *A. platys* and *A. phagocytophilum* morulae compared to whole blood. As these organisms are limited to platelets and leukocytes, this enriched fraction is cogently useful for the identification of cases of leucopenia and thrombocytopenia encountered as clinical sequelae [140].

For specific research, scanning electron microscopy, confocal microscopy, and transmission electron microscopy can be useful for the detection of these bacteria in ticks, mites, lice, and other invertebrates. Smears of tissue impressions from the liver, spleen, heart, lungs, kidneys, and/or blood vessels can be used during necropsy, especially for wild animal species [139,141].

### 9.2. Serology

Some companies provide commercial diagnostic kits for the serodiagnosis of anaplasmosis in animals and humans with variable accuracy. The IgG and IgM antibodies for *A. phagocytophilum* can be detected using a commercial kit based on IFAT “Fuller Laboratories” [143]. In addition, “SNAP 4Dx Plus,” a commercial test, allows for the detection of antibodies to *A. platys* and *A. phagocytophilum*, as well as other canine pathogens (*Ehrlichia canis* or *Ehrlichia ewingii*, *Borrelia burgdorferi*, and *Dirofilaria immitis*).

Likewise, the “Canine *Anaplasma* Antibody Test Kit” of VetScan^®^ (Abaxis) provides a rapid test thatqualitatively detects *A. platys* and/or *A. phagocytophilum* antibodies in dog serum or plasma. Similarly, “Anti-*Anaplasma phagocytophilum* ELISA Dog (IgG)” from EUROIMMUN (Medizinische Labordiagnostika AG) identifies seropositivity to *A. phagocytophilum* in dogs. Furthermore, the “Rapid *Anaplasma* Ab Test Kit” from the BIONOTE company is a commercial immunological test kit thatallows the qualitative chromatographic detection of the antibodies of *A. phagocytophilum* and *A. platys*.

Nevertheless, “MegaCorDiagnostik” performs immunochromatographic testing based on the lateral flow method, and “FASTest *Anaplasma*” provides qualitative discovery of *Anaplasma phagocytophilum* antibodies in dog and horse serum/plasma, which are commercially available for the convenient screening of anaplasmosis.

### 9.3. Molecular Detection

Nucleic acid detection methods, including conventional, nested and semi-nested PCR, real-time PCR, and LAMP (loop-mediated isothermal amplification), have been used for diagnosis. The *16S rRNA*, citrate synthase, heat shock, and major surface proteins (Msp1, Msp2, Msp4, and Msp5) are the most targeted genes for the molecular diagnosis of anaplasmosis [144].

Various high-performance single molecular and multiplex detection techniques with automation potential are in vogue. Whole blood containing ethylenediaminetetraacetic acid/citrate and buffy coat are good samples for diagnosis, while spleen samples are offered for the detection of carrier animals, especially in cases of wild animals [140,145,146]. Other types of samples, including the plasma/serum, lungs, liver, lymph nodes, skin, and bone marrow, have been used for screening [140,147,148,149]. For molecular diagnosis, multi copy genes are preferred over single copy genes. There is a growing trend to use fast and sensitive real-time assays over nested PCRs. Nonetheless, real-time screening assays yield short DNA products of less than 150 bp, yielding limited phylogenetical data. Sequencing and cross-matching are generally required for confirmation.

Different LAMP protocols have been developed for the identification of several *Anaplasma* species targeting the *msp1b*, *gltA*, *16S rRNA*, and *msp5* genes with variable detection limits. LAMP has the advantage of being a simple, robust, inexpensive, rapid, highly sensitive, and explicit diagnostic tool with low heat requirements, as well as options to use numerous primers [150]. For example, Lee and coworkers developed LAMP for the detection of *A. phagocytophilum* in dogs using the *gltA* gene, and this method was found to be more sensitive than nested PCR [151]. Likewise, Li and his colleagues developed a real-time LAMP for *A. platys* in dogs using citrate synthase gene sequences at 63 °C for 30 min. Uniform results and no cross-reactivity with other *Anaplasma*/*Ehrlichia* species were observed compared to nested PCR results [152].

Real-time molecular diagnostic methods have been developed for direct detection in blood, tissue, ticks/vectors that target multiple genes, which can be further used for taxonomic and phylogenetic studies. Whole genome sequencing of *A. phagocytophilum* and *A. platys* has been completed [34,35,153]. This will further contribute to the development of vaccines and diagnostic and control approaches for these important bacteria.

### 9.4. Isolation and In Vitro Cultivation

Isolation and *in vitro* culture are crucial, as all emerging bacteria have been grown on artificial media or cell lines. Indeed, ethical implications for animal use have led researchers to adapt the isolation and propagation of *Anaplasma* in cell culture lines derived from mammals on an uninterrupted basis. The HL-60 and THP-1 cells have been widely used [154,155]. Tick cell lines are alternative options for the in vitro cultivation of *Anaplasma* species [156]. Fresh infected blood from animals or humans is the best inoculum for in vitro propagation. For example, the cell line derived from embryos of *Ixodes scapularis* (IDE8, ISE6), *R. appendiculatus* (RAE25), *Dermacentor variabilis* (DVE1), as well as the cell lines *I. ricinus* IRE/CTVM19, IRE11, L610, and IRE/CTVM20, have been used for the culture and isolation of *A. phagocytophilum* [144,157]. Cells derived from *I. scapularis* (ISE6) are also used for culturing new isolates of *A. platys*-like bacteria [28].

## 10. Control

Usually, the control of anaplasmosis is difficult due to the existing antigenic/genetic diversity, the involvement of several hosts and multiple arthropod vectors, as well as different transmission potentials (biological, mechanical, and transplacental) [1]. In general, control measures include the control of arthropod vectors, host resistance and vaccination, sanitary/hygienic measures, and rarely chemoprophylaxis. However, the deterrence of tick infestation during periods of active transmission appears to be the best policy for the control of animal and human anaplasmosis [1].

### 10.1. Vector Control

Prevention strategies for common tick-borne diseases of domestic animals are based on the reduction of tick infestation using chemical acaricides [2]. Acaricidal treatment should be applied especially during the tick season. Biological tick control is gaining in importance as a striking approach to take, but it is generally difficult to achieve, since ticks have few natural enemies. Therefore, studies have focused on bacteria, entomopathogenic nematodes, and fungi [158,159]. However, the major concern is to establish sustainable biological control of ticks in natural habitats.

Tick vaccines are alternative control options against acaricides. The vaccination of animal–human populations at risk and/or the reservoir are important for limiting the distribution of tick-borne pathogens [160,161]. The development of combined vaccines targeting both pathogens transmitted by ticks and ticks themselves would be beneficial at large. There are various candidate proteins for a tick vaccine such as Bm86, Ba86, 64P, and RmAQP1. In addition, the salivary proteins Salp16 and Sialo L2 from *I. scapularis* protect the transmission of *A. phagocytophilum* infection [162].

Similarly, *I. ricinus* heme lipoprotein and uncharacterized secreted protein, as well asfive of the secreted proteins of *D. reticulatus* (glypican-like), which are involved in anion or sulfate exchangers, homophilic cell adhesion, subunit 3 of the signal peptidase complex, and other secreted proteins have been identified as the most effective vaccine candidates [163].

Subolesin is a protein that plays a role in reproduction, blood digestion, and development of ticks [164]. These types of vaccines cause disintegration of reproductive and embryonic tissues, causing sterility in male ticks, as well as degeneration of tick guts and salivary glands [165]. Tick vaccines are possible, cost-effective, and environmentally friendly methods compared to chemical control [44].

### 10.2. Vaccination against A. phagocytophilum and A. platys

Vaccination is the most effective and cheapest defense against anaplasmosis. It should be mentioned that the complete genome sequence of *A. phagocytophilum* and *A. platys* has been accomplished [34,35,153]. This can help to explore many new genes that could be potential candidates for vaccine manufacturing. There are approximately nine *Anaplasma* proteins that have immunogenic potential, namely, the Asp14, Asp55, Msp5, Msp2, AipA, OmpA, APH 0032, and APH 1384 antigens of the type IV secretion system of *A. phagocytophilum* [153,166,167,168,169].

### 10.3. Chemotherapeutic Use

*Anaplasma* bacteria are sensitive to antibiotics from the tetracycline group. Doxycycline is effective against human granulocytic anaplasmosis [11]. Similarly, doxycycline is also a useful chemotherapeutic agent for *A. platys* infection in dogs at a dose rate of 10 mg/kg body weight orally with or without dexamethasone (0.3 mg/kg IM daily) for 28 days [148]. In a situation of severe anemia, a blood transfusion is necessary.

Likewise, *A. phagocytophilum* infection in dogs can be treated with orbifloxacin at 5 mg/kg SC on day 1 and then orally on day 2 SID for two weeks. Other options that have proven effective for *A. phagocytophilum* infection in dogs include enrofloxacine (5 mg/kg SC, SID) and prednisolone (SID 1.5 mg/kg SC) on day 1, and orbifloxacin (4 mg/kg SC) and prednisolone (1 mg/kg SC) from days 2–8 [170]. Animals with severe anemia accompanied by debility should be hospitalized.

Concomitant infection with *A. platys* and/or *A. phagocytophilum* in dogs, as well as with *E. canis*, *Babesia vogeli, Borrelia burgdorferi, Hepatozoon canis* and/or *Leishmania infantum*, has been reported [5,171]. Concurrent infections may obscure epidemiology, alter treatment, and present an atypical clinical picture. The clinical veterinarian should keep this aspect of coinfection in mind when dealing with anaplasmosis cases in the clinic. A summary of the treatment protocols is presented in Table 4.

## 11. Conclusions

Concretely, the adoption of control strategies varies according to geo-ecological circumstances. Integrated control of major reservoirs/vectors/ticks and hygienic sanitary measures are key elements in reducing disease transmission. As there is greater risk of mortality in older animals than younger ones, thus these individuals/animals should be treated as a priority. Doxycycline with or without dexamethasone is a significant treatment for human and animal anaplasmosis. However, reducing high-risk tick contact activities in humans (such as gardening and hiking), careful blood transfusion, circumventing immunosuppression, recognizing reservoirs/vectors, and control ofvectorsare significant defense strategies against human anaplasmosis.

## Figures and Tables

**Table 1:** Classified and unclassified *Anaplasma* species infecting different cells, their vertebrate hosts, and their potential vectors.

*Anaplasma* Species	Infecting Cells	Vertebrate Hosts	Potential Vectors	References
*A. platys*	Platelets	Dogs and camels	*Rhipicephalus*	[22,23]
*A. phagocytophilum*	Granulocytes	Domestic and wild ruminants, horses, dogs, cats, rabbits, rodents, insectivores, wild swine, and humans	*Ixodes*, *Dermacentor,* *Hyalomma,* *Rhipicephalus*	[13]
*A. marginale*	Erythrocytes	Domestic ruminants	*Rhipicephalus, Ixodes*, *Dermacentor*	[24]
*A. centrale*	Erythrocytes	Domestic and wild ruminants	*Rhipicephalus, Ixodes*, *Haemaphysalis*	[1]
*A. ovis*	Erythrocytes	Domestic and wild ruminants and humans	*Rhipicephalus, Dermacentor,* *Hyalomma*	[1]
*A. bovis*	Monocytes	Domestic and wild ruminants and small mammals	*Haemaphysalis*, *Rhipicephalus*, *Amblyomma*	[25]
*A. capra*	Erythrocytes	Domestic and wild ruminants and humans	*Haemaphysalis*	[26,27]
*A. odocoilei*	Platelets	Wild ruminants	Not known	[28]
*Candidatus* A. camelii	Not known	Camels	Not known	[29]
*Candidatus* A. boleense	Not known	Not known	*Hyalomma*	[30]
*Candidatus* A. corsicanum	Not known	Domestic ruminants	Not known	[31]
*Candidatus* A. mediterraneum	Not known	Domestic ruminants	Not known	[31]
*Candidatus* A. sphenisci	Not known	African penguins	Not known	[32]
*Candidatus* A. rodmosense	Not known	Rodents	Not known	[33]

**Table 3:** Detection of *A. phagocytophilum* from the tissue or blood of domestic canids hosts *.

Domestic Canid	Countries (Regions)	Prevalences (%)	Methods(Target Genes)	References
Dog	Iraq	55.6	Blood smear	[113]
	Iran	2.0	PCR ^a^ (*msp4*)	[114]
	Mexico	27	PCR^a^ (16S rRNA)	[115]
	USA (California)	7.6	RT-PCR^b^ (*msp2*)	[116]
	Brazil	7.1	RT-PCR^b^ (*msp2*)	[117]
	USA (South)	2.1	ELISA^c^	[83]
	USA (Mid-Atlantic)	5.4	ELISA^c^	[83]
	USA (Northeast)	13	ELISA^c^	[83]
	USA (Midwest)	1.9	ELISA^c^	[83]
	USA (West)	2.0	ELISA^c^	[83]
	Canada	1.1	ELISA^c^	[83]
	Caribbean	3.4	ELISA^c^	[83]
	Sweden	17.0	IFAT^d^	[118]
	Colombia	1.1	PCR^a^ (16S rRNA)	[119]
	Costa Rica	0.3	PCR^a^ (16SrRNA, *groEL*)	[86]
	India	0.4	PCR^a^ (16S/18S rRNA)	[108]
	Turkey	4.0	nPCR^e^ (16S rRNA)	[120]

^a^ Polymerase chain reaction; ^b^ real-time polymerase chain reaction; ^c^ enzyme-linked immunosorbent assay; ^d^ indirect fluorescent antibody test; ^e^ nested polymerization chain reaction. * Detection of *A. phagocytophilum* from 1998 up to date.

**Table 4:** Summary of the clinical findings, diagnosis, and control of canine cyclic thrombocytopenia, and canine and human granulocytic anaplasmosis.

Disease	Clinical Findings	Diagnosis	Treatment	Control
Canine cyclic thrombocytopenia	Dogs usually remain asymptomatic; however, fever, lethargy, anorexia, weight loss, anemia, icterus, petechiae, nasal discharge, lymphadenopathy, and lymphadenomegaly may beobserved [5]	Stained blood smear, thrombocytopenia, serology, and PCR/DNA sequencing [5]	Doxycycline @5–10 mg kg^−1^ q12–24 h for 8–10 days orenrofloxacin @ 5mg kg^−1^, q12 h for 14–21 days [5]	Tick elimination, collar, pour-on or spot-on acaricidal products for *R. sanguineus sensu lato* ticks, knowledge of tick seasonality, andecology [5]
Canine granulocytic anaplasmosis	Non-specific signs, fever, anemia, anorexia, dullness, and thrombocytopenia [5]	Morulae in stained blood smear, thrombocytopenia, leucopenia, elevated liver enzymes, serology, andPCR/DNA sequencing [1,5]	Doxycycline 5mg/kg bid for 28 days [172]	Vector control, habitat modification, rearing tick-resistant breeds, and chemotherapy [5]
Human granulocyticanaplasmosis	Fever, headache, myalgias, and chills [123]	Morulae in stained blood smear, thrombocytopenia, leucopenia, elevated liver enzymes, serology/IFA, and PCR/DNA sequencing [95,173]	Doxycycline @ 100mg, orally, twice dailyfor 10–14 days or rifampicin @ 20 mgkg^−1^ day^−1^ orallyfor children, otherwise 300 mg orally, twice dailyfor 5–7 days [47]	Humans: Minimizing high-risk tick exposure activities (hiking, gardening, etc.), blood transfusion, immune suppression, identificationof reservoirs and vectors, and their control [1]

## Data Availability

The study did not report any data.

## References

[1] Atif F.A. (2016). Alpha proteobacteria of genus Anaplasma (Rickettsiales: Anaplasmataceae): Epidemiology and characteristics of Anaplasma species related to veterinary and public health importance. Parasitology.

[2] Ben Said M., Belkahia H., Messadi L. (2018). Anaplasma spp. in North Africa: A review on molecular epidemiology, associated risk factors and genetic characteristics. Ticks Tick Borne Dis..

[3] Stiller D., Crosbie P.R., Boyce W.M., Goff W.L. (1999). *Dermacentor hunteri* (Acari: Ixodidae): An experimental vector of *Anaplasma marginale* and *A. ovis* (Rickettsiales: Anaplasmataceae) to calves and sheep. J. Med. Entomol..

[4] Harvey J.W., Simpson C.F., Gaskin J.M. (1978). Cyclic thrombocytopenia induced by a Rickettsia-like agent in dogs. J. Infect. Dis..

[5] Sainz A., Roura X., Miro G., Estrada-Pena A., Kohn B., Harrus S., Solano-Gallego L. (2015). Guideline for veterinary practitioners on canine ehrlichiosis and anaplasmosis in Europe. Parasit. Vectors.

[6] Battilani M., De Arcangeli S., Balboni A., Dondi F. (2017). Genetic diversity and molecular epidemiology of Anaplasma. Infect. Genet. Evol..

[7] Chen S.M., Dumler J.S., Bakken J.S., Walker D.H. (1994). Identification of a granulocytotropic Ehrlichia species as the etiologic agent of human disease. J. Clin. Microbiol..

[8] Arraga-Alvarado C., Palmar M., Parra O., Salas P. (1999). Fine structural characterisation of a Rickettsia-like organism in human platelets from patients with symptoms of ehrlichiosis. J. Med. Microbiol..

[9] Maggi R.G., Mascarelli P.E., Havenga L.N., Naidoo V., Breitschwerdt E.B. (2013). Co-infection with *Anaplasma platys*, *Bartonella henselae* and *Candidatus* Mycoplasma haematoparvum in a veterinarian. Parasit. Vectors.

[10] Breitschwerdt E.B., Hegarty B.C., Qurollo B.A., Saito T.B., Maggi R.G., Blanton L.S., Bouyer D.H. (2014). Intravascular persistence of *Anaplasma platys*, *Ehrlichia chaffeensis*, and *Ehrlichia ewingii* DNA in the blood of a dog and two family members. Parasit. Vectors.

[11] CDC Centers for Disease Control and Prevention, National Center for Emerging and Zoonotic Infectious Diseases (NCEZID), Division of Vector-Borne Diseases (DVBD). https://www.cdc.gov/anaplasmosis/stats/index.html.

[12] Reppert E., Galindo R.C., Breshears M.A., Kocan K.M., Blouin E.F., de la Fuente J. (2013). Demonstration of transplacental transmission of a human isolate of *Anaplasma phagocytophilum* in an experimentally infected sheep. Transbound. Emerg. Dis..

[13] Stuen S., Granquist E.G., Silaghi C. (2013). *Anaplasma phagocytophilum*-a widespread multi-host pathogen with highly adaptive strategies. Front. Cell. Infect. Microbiol..

[14] Gordon W.S., Brownlee A., Wilson D.R., MacLeod J. (1932). Tick-Borne Fever (A hitherto undescribed disease of sheep). J. Comp. Path..

[15] MacLeod J., Gordon W.S. (1933). Studies on tick borne fever in sheep I. Transmission by the tick *Ixodes ricinus* and description of the disease produced. Parasitology.

[16] MacLeod J. (1936). Studies on tick-borne fever of sheep. 2. Experiment on transmission and distribution of the disease. Parasitology..

[17] Foggie A. (1949). Studies on tick-borne fever in sheep. J. Gen. Microbiol..

[18] Dumler J., Choi K., Garcia J., Barat N., Scorpio D., Garyu J., Grab D., Bakken J. (2005). Human Granulocytic Anaplasmosis and *Anaplasma phagocytophilum*. Emerg. Infect. Dis..

[19] Gribble D.H. (1969). Equine ehrlichiosis. J. Am. Vet. Med. Assoc..

[20] Lewis J.E., Huxsoll D.L., Ristic M., Johnson A.J. (1975). Experimentally induced infection of dogs, cats, and nonhuman primates with *Ehrlichia equi*, etiologic agent of equine ehrlichiosis. Am. J. Vet. Res..

[21] Bjöersdorff A., Bagert B., Massung R.F., Gusa A., Eliasson I. (2002). Isolation and characterization of two European strains of *Ehrlichia phagocytophila* of equine origin. Clin. Diagn. Lab. Immunol..

[22] Li H., Zheng Y., Ma L., Jia N., Jiang B., Jiang R., Huo Q., Wang Y., Liu H., Chu Y.L. (2015). Human infection with a novel tick-borne Anaplasma species in China: A surveillance study. Lancet Infect. Dis..

[23] Chandra S., Smith K., Alanazi A.D., Alyousif M.S., Emery D., Šlapeta J. (2019). *Rhipicephalus sanguineus* sensu lato from dogs and dromedary camels in Riyadh, Saudi Arabia: Low prevalence of vector-borne pathogens in dogs detected using multiplexed tandem PCR panel. Folia Parasitol..

[24] Atif F.A. (2015). *Anaplasma marginale* and *Anaplasma phagocytophilum*: Rickettsiales pathogens of veterinary and public health significance. Parasitol. Res..

[25] Kawahara M., Rikihisa Y., Lin Q., Isogai E., Tahara K., Itagaki A., Hiramitsu Y., Tajima T. (2006). Novel genetic variants of *Anaplasma phagocytophilum*, *Anaplasma bovis*, *Anaplasma centrale*, and a novel Ehrlichia sp. in wild deer and ticks on two major islands in Japan. Appl. Environ. Microbiol..

[26] Yang J., Liu Z., Niu Q., Liu J., Han R., Liu G., Shi Y., Luo J., Yin H. (2016). Molecular survey and characterization of a novel Anaplasma species closely related to *Anaplasma capra* in ticks, northwestern China. Parasit. Vectors.

[27] Peng Y., Lu C., Yan Y., Shi K., Chen Q., Zhao C., Wang R., Zhang L., Jian F., Ning C. (2021). The first detection of *Anaplasma capra*, an emerging zoonotic Anaplasma sp., in erythrocytes. Emerg. Microbes. Infect..

[28] Tate C.M., Howerth E.W., Mead D.G., Dugan V.G., Luttrell M.P., Sahora A.I., Munderloh U.G., Davidson W.R., Yabsley M.J. (2013). *Anaplasma odocoilei* sp. nov. (family Anaplasmataceae) from white-tailed deer (*Odocoileus virginianus*). Ticks Tick Borne Dis..

[29] Lbacha A.H., Zouagui Z., Alali S., Rhalem A., Petit E., Ducrotoy M.J., Boulouis H.-J., Maillard R. (2017). “*Candidatus* anaplasma camelii” in one-humped camels (Camelus dromedarius) in Morocco: A novel and emerging Anaplasma species?. Infect. Dis. Poverty.

[30] Gofton A.W., Hair S., Ryan U., Irwin P. (2018). Initial detection of *Ehrlichia mineirensis* and ‘ *Candidatus* Anaplasma boleense’ in an Australian steer. Mol. Genet. Genom..

[31] Dahmani M., Davoust B., Sambou M., Bassene H., Scandola P., Ameur T., Raoult D., Fenollar F., Mediannikov O. (2019). Molecular investigation and phylogeny of species of the Anaplasmataceae infecting animals and ticks in Senegal. Parasit. Vectors.

[32] Vanstreels R.E.T., Yabsley M.J., Parsons N.J., Swanepoel L., Pistorius P.A. (2018). A novel candidate species of Anaplasma that infects avian erythrocytes. Parasit. Vectors.

[33] Guo W.P., Tian J.H., Lin X.D., Ni X.B., Chen X.P., Liao Y., Yang S.Y., Dumler J.S., Holmes E.C., Zhang Y.Z. (2016). Extensive genetic diversity of Rickettsiales bacteria in multiple mosquito species. Sci. Rep..

[34] Diaz-Sanchez S., Hernández-Jarguín A., de Mera I.G.F., Alberdi P., Zweygarth E., Gortazar C., de la Fuente J. (2018). Draft genome sequences of *Anaplasma phagocytophilum*, *A. marginale*, and *A. ovis* Isolates from different hosts. Genome Announc..

[35] Llanes A., Rajeev S. (2020). First whole genome sequence of *Anaplasma platys*, an obligate intracellular rickettsial pathogen of dogs. Pathogens.

[36] Chochlakis D., Ioannou I., Tselentis Y., Psaroulaki A. (2010). Human anaplasmosis and *Anaplasma ovis* variant. Emerg. Infect. Dis..

[37] Rar V., Golovljova I. (2011). Anaplasma, Ehrlichia, and “*Candidatus* Neoehrlichia” bacteria, pathogenicity, biodiversity, and molecular genetic characteristics, a review. Infect. Genet. Evol..

[38] Weisburg W.G., Barns S.M., Pelletier D.A., Lane D.J. (1991). 16S ribosomal DNA amplification for phylogenetic study. J. Bacteriol..

[39] Sumner J.W., Nicholson W.L., Massung R.F. (1997). PCR amplification and comparison of nucleotide sequences from the groESL heat shock operon of Ehrlichia species. J. Clin. Microbiol..

[40] Dumler J.S., Barbet A.F., Bekker C.P., Dasch G.A., Palmer G.H., Ray S.C., Rikihisa Y., Rurangirwa F.R. (2001). Reorganization of genera in the families Rickettsiaceae and Anaplasmataceae in the order Rickettsiales: Unification of some species of Ehrlichia with Anaplasma, Cowdria with Ehrlichia and Ehrlichia with Neorickettsia, descriptions of six new species combinations and designation of Ehrlichia equi and ‘HGE agent’ as subjective synonyms of *Ehrlichia phagocytophila*. Int. J. Syst. Evol. Microbiol..

[41] Carrade D.D., Foley J.E., Borjesson D.L., Sykes J.E. (2009). Canine granulocytic anaplasmosis: A review. J. Vet. Intern. Med..

[42] Berger S. (2021). Anaplasmosis, Global Status.

[43] Lima M.L., Soares P.T., Ramos C.A., Araújo F.R., Ramos R.A., Souza I.I., Faustino M.A., Alves L.C. (2010). Molecular detection of Anaplasma platys in a naturally-infected cat in Brazil. Braz. J. Microbiol..

[44] Kocan K.M., de la Fuente J., Guglielmone A.A., Melendez R.D. (2003). Antigens and Alternatives for Control of Anaplasma marginale Infection in Cattle. Clin. Microbiol. Rev..

[45] Arraga-Alvarado C.M., Qurollo B.A., Parra O.C., Berrueta M.A., Hegarty B.C., Breitschwerdt E.B. (2014). Case report: Molecular evidence of Anaplasma platys infection in two women from Venezuela. Am. J. Trop. Med. Hyg..

[46] Sainz A., Amusategui I., Tesouro M.A. (1999). Ehrlichia platys infection and disease in dogs in Spain. J. Vet. Diagn. Investig..

[47] Suksawat J., Xuejie Y., Hancock S.I., Hegarty B.C., Nilkumhang P., Breitschwerdt E.B. (2001). Serologic and molecular evidence of coinfection with multiple vector-borne pathogens in dogs from Thailand. J. Vet. Intern. Med..

[48] Sparagano O.A.E., Vos A.P.d., Paoletti B., Camma C., Santis P.d., Otranto D., Giangaspero A. (2003). Molecular detection of *Anaplasma platys* in dogs using polymerase chain reaction and reverse line blot hybridization. J. Vet. Diagn. Investig..

[49] Brown R.N., Lane R., Dennis D.T. (2005). Geographic distributions of tick-borne diseases and their Vectors. Tick Borne Dis. Humans.

[50] Aguirre E., Tesouro M.A., Ruiz L., Amusategui I., Sainz A. (2006). Genetic characterization of Anaplasma (Ehrlichia) platys in dogs in Spain. J. Vet. Med. Ser. B..

[51] Melo A.L.T., Witter R., Martins T.F., Pacheco T.A., Alves A.S., Chitarra C.S., Aguiar D.M. (2016). A survey of tick-borne pathogens in dogs and their ticks in the Pantanal biome, Brazil. Med. Vet. Entomol..

[52] Sudan V., Sharma R.L., Borah M.K. (2014). Subclinical anaplasmosis in camel (*Camelus dromedarius*) and its successful therapeutic management. J. Parasit. Dis..

[53] Lorusso V., Wijnveld M., Majekodunmi A.O., Dongkum C., Fajinmi A., Dogo A.G., Thrusfield M., Mugenyi A., Vaumourin E., Igweh A.C. (2016). Tick-borne pathogens of zoonotic and veterinary importance in Nigerian cattle. Parasit. Vectors.

[54] Bahrami S., Hamidinejat H., Tafreshi A.R.G. (2018). First molecular detection of *Anaplasma Phagocytophilum* in Dromedaries ( Camelus Dromedarius). J. Zoo Wildl. Med..

[55] Woldehiwet Z. (2006). *Anaplasma phagocytophilum* in ruminants in Europe. Ann. N. Y. Acad. Sci..

[56] Dahlgren F.S., Mandel E.J., Krebs J.W., Massung R.F., McQuiston J.H. (2011). Increasing incidence of *Ehrlichia chaffeensis* and *Anaplasma phagocytophilum* in the United States, 2000–2007. Am. J. Trop. Med. Hyg..

[57] Bakken J.S., Dumler J.S. (2015). Human granulocytic anaplasmosis. Infect. Dis. Clin..

[58] Fishbein D.B., Raoult D. (1992). A cluster of Coxiella burnetii infections associated with exposure to vaccinated goats and their unpasteurized dairy products. Am. J. Trop. Med. Hyg..

[59] Dumler J.S., Bakken J.S. (1998). Human ehrlichioses: Newly recognized infections transmitted by ticks. Annu. Rev. Med..

[60] Blanco J.R., Oteo J.A. (2002). Human granulocytic ehrlichiosis in Europe. Clin. Microbiol. Infect..

[61] Woldehiwet Z. (2010). The natural history of *Anaplasma phagocytophilum*. Vet. Parasitol..

[62] Do T., Phoosangwalthong P., Kamyingkird K., Kengradomkij C., Chimnoi W., Inpankaew T. (2021). Molecular detection of tick-borne pathogens in stray dogs and *Rhipicephalus sanguineus* sensu lato ticks from Bangkok, Thailand. Pathogens.

[63] Piratae S., Senawong P., Chalermchat P., Harnarsa W., Sae-Chue B. (2019). Molecular evidence of *Ehrlichia canis* and *Anaplasma platys* and responses in naturally infected dogs in Kalasin, Thailand. Vet. World..

[64] Buddhachat K., Meerod T., Pradit W.P.S., Chomdej S., Nganvongpanit K. (2020). Simultaneous differential detection of canine blood parasites: Multiplex high-resolution melting analysis (mHRM). Ticks Tick Borne Dis..

[65] Alhassan A., Hove P., Sharma B., Matthew-Belmar V., Karasek I., Lanza-Perea M., Werners A.H., Wilkerson M.J., Ganta R.R. (2021). Molecular detection and characterization of *Anaplasma platys* and *Ehrlichia canis* in dogs from the Caribbean. Ticks Tick Borne Dis..

[66] Wilkerson M.J., Black K.E., Lanza-Perea M., Sharma B., Gibson K., Stone D.M., George A., Nair A.D., Ganta R.R. (2017). Initial development and preliminary evaluation of a multiplex bead assay to detect antibodies to *Ehrlichia canis*, *Anaplasma platys*, and *Ehrlichia chaffeensis* outer membrane peptides in naturally infected dogs from Grenada, West Indies. J. Vet. Diagn. Investig..

[67] Sharma B., Ganta R., Stone D., Alhassan A., Lanza-Perea M., Matthew Belmar V., Karasek I., Cooksey E.M., Butler C., Gibson K. (2021). Development of a multiplex pcr and magnetic dna capture assay for detecting six species pathogens of the genera Anaplasma and Ehrlichia in canine, bovine, caprine and ovine blood samples from Grenada, West Indies. Pathogens.

[68] Georges K., Ezeokoli C.D., Newaj-Fyzul A., Campbell M., Mootoo N., Mutani A., Sparagano O.A. (2008). The application of PCR and reverse line blot hybridization to detect arthropod-borne hemopathogens of dogs and cats in Trinidad. Ann. N. Y. Acad. Sci..

[69] Ghauri H.N., Ijaz M., Ahmed A., Muhammad Naveed M.U.A., Nawab Y., Javed M.U., Ghaffar A. (2021). Molecular investigation and phylogenetic analysis of anaplasmosis in dogs. J. Parasitol..

[70] Pérez-Macchi S., Pedrozo R., Bittencourt P., Müller A. (2019). Prevalence, molecular characterization and risk factor analysis of *Ehrlichia canis* and *Anaplasma platys* in domestic dogs from Paraguay. Comp. Immunol. Microbiol. Infect. Dis..

[71] Pesapane R., Foley J., Thomas R., Castro L.R. (2019). Molecular detection and characterization of *Anaplasma platys* and *Ehrlichia canis* in dogs from northern Colombia. Vet. Microbiol..

[72] Kontos V.I., Papadopoulos O., French T.W. (1991). Natural and experimental canine infections with a Greek strain of *Ehrlichia platys*. Vet. Clin. Pathol..

[73] Faizal M.D., Haryanto A., Tjahajati I. (2019). Diagnosis and molecular characterization of *Anaplasma platys* in dog patients in Yogyakarta area, Indonesia. Indones. J. Biotechnol..

[74] Götsch S., Leschnik M., Duscher G., Burgstaller J.P., Wille-Piazzai W., Joachim A. (2009). Ticks and haemoparasites of dogs from Praia, Cape Verde. Vet. Parasitol..

[75] de Caprariis D., Dantas-Torres F., Capelli G., Mencke N., Stanneck D., Breitschwerdt E.B., Otranto D. (2011). Evolution of clinical, haematological and biochemical findings in young dogs naturally infected by vector-borne pathogens. Vet. Microbiol..

[76] Ramos R.A., Latrofa M.S., Giannelli A., Lacasella V., Campbell B.E., Dantas-Torres F., Otranto D. (2014). Detection of *Anaplasma platys* in dogs and *Rhipicephalus sanguineus* group ticks by a quantitative real-time PCR. Vet. Parasitol..

[77] Dyachenko V., Pantchev N., Balzer H.J., Meyersen A., Straubinger R.K. (2012). First case of Anaplasma platys infection in a dog from Croatia. Parasite Vector..

[78] Barker E.N., Langton D.A., Helps C.R., Brown G., Malik R., Shaw S.E., Tasker S. (2012). Haemoparasites of free-roaming dogs associated with several remote Aboriginal communities in Australia. BMC Vet. Res..

[79] Hii S.F., Traub R.J., Thompson M.F., Henning J., O’Leary C.A., Burleigh A., Kopp S.R. (2015). Canine tick-borne pathogens and associated risk factors in dogs presenting with and without clinical signs consistent with tick-borne diseases in northern Australia. Aust. Vet. J..

[80] Andersson M., Turcitu M.A., Stefanache M., Tamba P., Barbuceanu F., Chitimia L. (2013). First evidence of *Anaplasma platys* and Hepatozoon canis co-infection in a dog from Romania-a case report. Ticks TickBorne Dis..

[81] Kelly P.J., Lucas H., Eremeeva M.E., Dirks K.G., Rolain J.M., Yowell C., Thomas R., Douglas T., Dasch G.A., Raoult D. (2010). Rickettsia felis, West Indies. Emerg. Infect. Dis..

[82] Wei L., Kelly P., Ackerson K., Zhang J., El-Mahallawy H.S., Kaltenboeck B., Wang C. (2014). First report of *Babesia gibsoni* in Central America and survey for vector-borne infections in dogs from Nicaragua. Parasit. Vectors.

[83] Qurollo B.A., Chandrashekar R., Hegarty B.C., Beall M.J., Stillman B.A., Liu J., Thatcher B., Pultorak E., Cerrito B., Walsh M. (2014). A serological survey of tick-borne pathogens in dogs in North America and the Caribbean as assessed by *Anaplasma phagocytophilum*, *A. platys*, *Ehrlichia canis*, *E. chaffeensis*, *E. ewingii*, and *Borrelia burgdorferi* species-specific peptides. Infect. Ecol. Epidemiol..

[84] Almazán C., González-Álvarez V.H., de Mera I.G.F., Cabezas-Cruz A., Rodríguez-Martínez R., de la Fuente J. (2016). Molecular identification and characterization of *Anaplasma platys* and *Ehrlichia canis* in dogs in Mexico. Ticks TickBorne Dis..

[85] Aktas M., Altay K., Dumanli N., Kalkan A. (2009). Molecular detection and identification of Ehrlichia and Anaplasma species in ixodid ticks. Parasitol. Res..

[86] Bonilla M.C., Campos-Calderón L., Jiménez-Rocha A.E., Romero-Zúñiga J.J., Alberti A., Zobba R., Dolz G. (2017). Characterization of Anaplasma spp. infection in dogs from Costa Rica. Vet. Parasitol. Reg. Stud. Rep..

[87] Soares R., Ramos C.A., Pedroso T., Babo-Terra V., Cleveland H., Araújo F. (2017). Molecular survey of *Anaplasma platys* and *Ehrlichia canis* in dogs from Campo Grande, Mato Grosso do Sul, Brazil. Acad. Bras. Cienc..

[88] da Silva G.C., Benitez A., Girotto A., Taroda A., Vidotto M.C., Garcia J.L., de Freitas J.C., Arlington S.H., Vidotto O. (2012). Occurrence of *Ehrlichia canis* and *Anaplasma platys* in household dogs from northern Parana. Rev. Bras. Parasitol. Vet..

[89] Lasta C.S., dos Santos A.P., Messick J.B., Oliveira S.T., Biondo A.W., Vieira R.F., Dalmolin M.L., González F.H. (2013). Molecular detection of *Ehrlichia canis* and *Anaplasma platys* in dogs in Southern Brazil. Rev. Bras. Parasitol. Vet..

[90] McCown M.E., Alleman A., Sayler K.A., Chandrashekar R., Thatcher B., Tyrrell P., Stillman B., Beall M., Barbet A.F. (2014). Point prevalence survey for tick-borne pathogens in military working dogs, shelter animals, and pet populations in northern Colombia. J. Spec. Oper. Med..

[91] Zaid T., Ereqat S., Nasereddin A., Al-Jawabreh A., Abdelkader A., Abdeen Z. (2019). Molecular characterization of AnaplasmaandEhrlichiain ixodid ticks and reservoir hosts from Palestine: A pilot survey. Vet. Med. Sci..

[92] Yang B., Ye C., Sun E., Wen Y., Qian D., Sun H. (2020). First molecular evidence of *Anaplasma* spp. co-infection in stray dogs from Anhui, China. Acta. Trop..

[93] Cicuttin G., Boeri E., Beltrán F., Gury D., Federico E. (2013). Molecular detection of Neorickettsiaristicii in Brazilian free-tailed bats (Tadaridabrasiliensis) from Buenos Aires, Argentina. Pesq. Vet. Bras..

[94] Springer A., Montenegro V., Schicht S., Wölfel S., Schaper S., Chitimia-Dobler L., Siebert S., Strube C. (2018). Detection of *Rickettsia monacensis* and *Rickettsia amblyommatis* in ticks collected from dogs in Costa Rica and Nicaragua. Ticks Tick Borne Dis..

[95] Hmoon M.M., Htun L.L., Thu M.J., Chel H.M., Thaw Y.N., Win S.Y., Chan Soe N., Khaing Y., Thein S.S., Bawm S. (2021). Molecular prevalence and identification of *Ehrlichia canis* and *Anaplasma platys* from Dogs in Nay Pyi Taw Area, Myanmar. Vet. Med. Int..

[96] Chatanga E., Kainga H., Razemba T., Ssuna R., Swennen L., Hayashida K., Sugimoto C., Katakura K., Nonaka N., Nakao R. (2021). Molecular detection and characterization of tick-borne hemoparasites and Anaplasmataceae in dogs in major cities of Malawi. Parasitol. Res..

[97] Jimenez I.A., Vega Mariño P.A., Stapleton G.S., Prieto J.B., Bowman D.D. (2020). Canine vector-borne disease in domestic dogs on Isla Santa Cruz, Galápagos. Vet. Parasitol. Reg. Stud. Rep..

[98] Alanazi A., Nguyen V., Alyousif M., Manoj R., Alouffi A., Donato R., Sazmand A., Mendoza-Roldan J., Torres F., Otranto D. (2020). Ticks and associated pathogens in camels (Camelus dromedarius) from Riyadh Province, Saudi Arabia. Parasit. Vectors.

[99] Diakou A., Di Cesare A., Morelli S., Colombo M., Halos L., Simonato G., Tamvakis A., Beugnet F., Paoletti B., Traversa D. (2019). Endoparasites and vector-borne pathogens in dogs from Greek islands: Pathogen distribution and zoonotic implications. PLoS Negl. Trop. Dis..

[100] Licari E., Takács N., Solymosi N., Farkas R. (2017). First detection of tick-borne pathogens of dogs from Malta. Ticks TickBorne Dis..

[101] Starkey L.A., Newton K., Brunker J., Crowdis K., Edourad E., Meneus P., Little S.E. (2016). Prevalence of vector-borne pathogens in dogs from Haiti. Vet. Parasitol..

[102] Huggins L.G., Colella V., Koehler A.V., Schunack B., Traub R.J. (2021). A multipronged next-generation sequencing metabarcoding approach unearths hyperdiverse and abundant dog pathogen communities in Cambodia. Transbound. Emerg. Dis..

[103] Proboste T., Kalema-Zikusoka G., Altet L., Solano-Gallego L., Fernández de Mera I.G., Chirife A.D., Muro J., Bach E., Piazza A., Cevidanes A. (2015). Infection and exposure to vector-borne pathogens in rural dogs and their ticks, Uganda. Parasit. Vectors.

[104] Hamel D., Shukullari E., Rapti D., Silaghi C., Pfister K., Rehbein S. (2016). Parasites and vector-borne pathogens in client-owned dogs in Albania. Blood pathogens and seroprevalences of parasitic and other infectious agents. Parasitol. Res..

[105] Kamani J., Morick D., Mumcuoglu K., Harrus S. (2013). Prevalence and diversity of Bartonella species in commensal rodents and ectoparasites from Nigeria, West Africa. PLoS Neglect. Trop. Dis..

[106] Alho A.M., Lima C., Latrofa M.S., Colella V., Ravagnan S., Capelli G., Madeira de Carvalho L., Cardoso L., Otranto D. (2017). Molecular detection of vector-borne pathogens in dogs and cats from Qatar. Parasit. Vectors.

[107] Modarelli J.J., Tomeček J.M., Piccione J., Ferro P.J., Esteve-Gasent M.D. (2019). Molecular prevalence and ecoregion distribution of select tick-borne pathogens in Texas dogs. Transbound. Emerg. Dis..

[108] Manoj R., Iatta R., Latrofa M.S., Capozzi L., Raman M., Colella V., Otranto D. (2020). Canine vector-borne pathogens from dogs and ticks from Tamil Nadu, India. Acta. Trop..

[109] Motoi Y., Satoh H., Inokuma H., Kiyuuna T., Muramatsu Y., Ueno H., Morita C. (2001). First detection of *Ehrlichia platys* in dogs and ticks in Okinawa, Japan. Microbiol. Immunol..

[110] Baldridge G.D., Scoles G., Burkhardt N.Y., Schloeder B., Kurtti T.J., Munderloh U.G. (2009). Transovarial transmission of Francisella-like endosymbionts and *Anaplasma phagocytophilum* variants in *Dermacentor albipictus* (Acari: Ixodidae). J. Med. Entomol..

[111] Dugat T., Lagrée A.C., Maillard R., Boulouis H.J., Haddad N. (2015). Opening the black box of *Anaplasma phagocytophilum* diversity: Current situation and future perspectives. Front. Cell. Infect. Microbiol..

[112] Fine A.B., Sweeney J.D., Nixon C.P., Knoll B.M. (2016). Transfusion-transmitted anaplasmosis from a leukoreduced platelet pool. Transfusion.

[113] Ahmed S.S., Khalaf J.M. (2020). First identification of *Anaplasma platys* and *Anaplasma phagocytophlium* in the blood of dogs in Baghdad Governorate. Plant Arch..

[114] Yousefi A.M.R.C., Golmohammadi A., Azami S. (2019). Molecular detection of *Anaplasma Phagocytophilum* as a zoonotic agent in owned and stray dogs in Tehran, Iran. Arch. Razi. Inst..

[115] Rojero-Vázquez E., Gordillo-Pérez G., Weber M. (2017). Infection of *Anaplasma phagocytophilum* and Ehrlichia spp. in opossums and dogs in Campeche, Mexico: The role of tick infestation. Front. Ecol. Evol..

[116] Henn J.B., Gabriel M.W., Kasten R.W., Brown R.N., Theis J.H., Foley J.E., Chomel B.B. (2007). Gray foxes (*Urocyon cinereoargenteus*) as a potential reservoir of a *Bartonella clarridgeiae*-like bacterium and domestic dogs as part of a sentinel system for surveillance of zoonotic arthropod-borne pathogens in northern California. J. Clin. Microbiol..

[117] Santos H.A., Pires M.S., Vilela J.A., Santos T.M., Faccini J.L., Baldani C.D., Thomé S.M., Sanavria A., Massard C.L. (2011). Detection of *Anaplasma phagocytophilum* in Brazilian dogs by real-time polymerase chain reaction. J. Vet. Diagn. Investing..

[118] Elfving K., Malmsten J., Dalin A.M., Nilsson K. (2015). Serologic and Molecular Prevalence of *Rickettsia helvetica* and *Anaplasma phagocytophilum* in Wild Cervids and Domestic Mammals in the Central Parts of Sweden. Vector Borne Zoonotic Dis..

[119] Vargas-Hernandez G., André M.R., Cendales D.M., de Sousa K.C.M., Gonçalves L.R., Rondelli M.C.H., Machado R.Z., Tinucci-Costa M. (2016). Molecular detection of Anaplasma species in dogs in Colombia. Rev. Bras. Parasitol. Vet..

[120] Çetinkaya H., Matur E., Akyazi İ., Ekiz E.E., Aydin L., Toparlak M. (2016). Serological and molecular investigation of Ehrlichia spp. and Anaplasma spp. in ticks and blood of dogs, in the Thrace Region of Turkey. Ticks Tick Borne Dis..

[121] Rikihisa Y. (1991). The tribe Ehrlichieae and ehrlichial diseases. Clin. Microbiol. Rev..

[122] Latrofa M.S., Dantas-Torres F., deCaprariis D., Cantacessi C., Capelli G., Lia R.P., Breitschwerdt E.B., Otranto D. (2016). Vertical transmission of *Anaplasma platys* and *Leishmania infantum* in dogs during the first half of gestation. Parasit. Vector..

[123] Matei I.A., D’Amico G., Yao P.K., Ionică A.M., Kanyari P.W.N., Daskalaki A.A., Dumitrache M.O., Sándor A.D., Gherman C.M., Qablan M. (2016). Molecular detection of *Anaplasma platys* infection in free-roaming dogs and ticks from Kenya and Ivory Coast. Parasit. Vectors.

[124] Stuen S., Okstad W., Sagen A.M. (2018). Intrauterine transmission of *Anaplasma phagocytophilum* in persistently infected lambs. Vet. Sci..

[125] Villar M., López V., Ayllón N., Cabezas-Cruz A., López J.A., Vázquez J., Alberdi P., de la Fuente J. (2016). The intracellular bacterium *Anaplasma phagocytophilum* selectively manipulates the levels of vertebrate host proteins in the tick vector Ixodes scapularis. Parasit. Vectors.

[126] Snellgrove A.N., Krapiunaya I., Ford S.L., Stanley H.M., Wickson A.G., Hartzer K.L., Levin M.L. (2020). Vector competence of *Rhipicephalus sanguineus* sensu stricto for Anaplasma platys. Ticks Tick Borne Dis..

[127] De Tommasi A.S., Baneth G., Breitschwerdt E.B., Stanneck D., Dantas-Torres F., Otranto D., de Caprariis D. (2014). Anaplasmaplatys in bone marrow megakaryocytes of young dogs. J. Clin. Microbial..

[128] Rikihisa Y. (2011). Mechanisms of obligatory intracellular infection with *Anaplasma phagocytophilum*. Clin. Microbiol. Rev..

[129] Bradfield J.F., Vore S.J., Pryor W.H.J. (1996). *Ehrlichia platys* infection in dogs. Lab. Anim. Sci..

[130] Bouzouraa T., René-Martellet M., Chêne J., Attipa C., Lebert I., Chalvet-Monfray K., Cadoré J.L., Halos L., Chabanne L. (2016). Clinical and laboratory features of canine *Anaplasma platys* infection in 32 naturally infected dogs in the Mediterranean basin. Ticks Tick Borne Dis..

[131] Kohn B., Galke D., Beelitz P., Pfister K. (2008). Clinical features of canine granulocytic anaplasmosis in 18 naturally infected dogs. J. Vet. Intern. Med..

[132] Nair A.D., Cheng C., Ganta C.K., Sanderson M.W., Alleman A.R., Munderloh U.G., Ganta R.R. (2016). Comparative experimental infection study in dogs with *Ehrlichia canis*, *E. chaffeensis*, *Anaplasma platys* and *A. phagocytophilum*. PLoS ONE.

[133] Bjöersdorff A., Svendenius L., Owens J.H., Massung R.F. (1999). Feline granulocytic ehrlichiosis—a report of a new clinical entity and characterisation of the infectious agent. J. Small Anim. Pract..

[134] Savidge C., Ewing P., Andrews J., Aucoin D., Lappin M.R., Moroff S. (2016). *Anaplasma phagocytophilum* infection of domestic cats: 16 cases from the northeastern USA. J. Feline Med. Surg..

[135] Sim R.R., Joyner P.H., Padilla L.R., Anikis P., Aitken-Palmer C. (2017). Clinical disease associated with *Anaplasma phagocytophilum* infection in captive Przewalski’s horses (Equus ferus przewalskii). J. Zoo Wildl. Med..

[136] Ismail N., Bloch K.C., McBride J.W. (2010). Human ehrlichiosis and anaplasmosis. Clin. Lab. Med..

[137] El-Khoury L., Furie R. (2019). Inflammatory arthritis: A unique presentation of human anaplasmosis. Clin. Rheumatol..

[138] Palmer G.H., Abbott J.R., French D.M., McElwain T.F. (1998). Persistence of *Anaplasma ovis* infection and conservation of the msp-2 and msp-3 multigene families within the genus Anaplasma. Infect. Immun..

[139] Kocan K.M., de la Fuente J., Blouin E.F., Coetzee J.F., Ewing S.A. (2010). The natural history of Anaplasma marginale. Vet. Parasitol..

[140] Eddlestone S.M., Gaunt S.D., Neer T.M., Boudreaux C.M., Gill A., Haschke E., Corstvet R.E. (2007). PCR detection of *Anaplasma platys* in blood and tissue of dogs during acute phase of experimental infection. Exp. Parasitol..

[141] OIE (2015). Bovine Anaplasmosis. Manual of Diagnostic Tests and Vaccines for Terestrial Animals.

[142] Choi S., Cho Y.U., Kim S.H. (2019). Morulae in neutrophils: A diagnostic clue for human granulocytic anaplasmosis. IDcases.

[143] Tokarz R., Tagliafierro T., Cucura D.M., Rochlin I., Sameroff S., Lipkin W.I. (2017). Detection of *Anaplasma phagocytophilum*, *Babesia microti*, *Borrelia burgdorferi*, *Borrelia miyamotoi* and Powassan virus in ticks by a multiplex real-time reverse transcription-PCR assay. mSphere.

[144] Silaghi C., Santos A.S., Gomes J., Christova I., Matei I.A., Walder G., Domingos A., Bell-Sakyi L., Sprong H., von Loewenich F.D. (2017). Guidelines for the direct detection of Anaplasma spp. in diagnosis and epidemiological studies. Vector Borne Zoonotic Dis..

[145] Hebels D.G., van Herwijnen M.H., Brauers K.J., de Kok T.M., Chalkiadaki G., Kyrtopoulos S.A., Kleinjans J.C. (2014). Elimination of heparin interference during microarray processing of fresh and biobank-archived blood samples. Environ. Mol. Mutagen..

[146] Sanchez-Fito M.T., Oltra E. (2015). Optimized treatment of heparinized blood fractions to make them suitable for analysis. Biopreserv. Biobank..

[147] Massung R.F., Slater K., Owens J.H., Nicholson W.L., Mather T.N., Solberg V.B., Olson J.G. (1998). Nested PCR assay for detection of granulocytic ehrlichiae. J. Clin. Microbiol..

[148] Gaunt S.D., Beall M.J., Stillman B.A., Lorentzen L., Diniz P.P.V.P., Chandrashekar R., Breitschwerdt E.B. (2010). Experimental infection and co-infection of dogs with *Anaplasma platys* and *Ehrlichia canis*: Hematologic, serologic and molecular findings. Parasit. Vector..

[149] Szekeres S., Coipan C.E., Rigó K., Majoros G., Jahfari S., Sprong H., Földvári G. (2015). Candidatus Neoehrlichia mikurensis and *Anaplasma phagocytophilum* in natural rodent and tick communities in Southern Hungary. Ticks TickBorne Dis..

[150] Njiru Z.K. (2012). Loop-mediated isothermal amplification technology: Towards point of care diagnostics. PLoS Negl. Trop. Dis..

[151] Lee C., Lin Y., Tsang C., Chung Y. (2012). A loop-mediated isothermal amplification (LAMP) assay for rapid detection of *Anaplasma phagocytophilum* infection in dogs. Turk. J. Vet. Anim. Sci..

[152] Li H.T., Sun L.S., Chen Z.M., Hu J.S., Ye C.D., Jia K., Wang H., Yuan L.G., Zhang G.H., Li S. (2014). Detection of Anaplasma platys in dogs using real-time loop-mediated isothermal amplification. Vet. J..

[153] Nelson C.M., Herron M.J., Felsheim R.F., Schloeder B.R., Grindle S.M., Chavez A.O., Kurtti T.J., Munderloh U.G. (2008). Whole genome transcription profiling of Anaplasma phagocytophilum in human and tick host cells by tiling array analysis. BMC Genomics.

[154] Munderloh U.G., Lynch M.J., Herron M.J., Palmer A.T., Kurtti T.J., Nelson R.D., Goodman J.L. (2004). Infection of endothelial cells with *Anaplasma marginale* and *A. phagocytophilum*. Vet. Microbiol..

[155] Shimada M., Takamoto N., Su H., Sasahara H., Shimamura Y., Ando S., Ohashi N. (2018). Predomination shift of different P44-expressing *Anaplasma phagocytophilum* in infected HL-60, THP-1, NB4, and RF/6A cell lines. Jpn. J. Infect. Dis..

[156] Massung R.F., Levin M.L., Munderloh U.G., Silverman D.J., Lynch M.J., Gaywee J.K., Kurtti T.J. (2007). Isolation and propagation of the Ap-Variant 1 strain of *Anaplasma phagocytophilum* in a tick cell line. J. Clin. Microbiol..

[157] Bell-Sakyi L., Darby A., Baylis M., Makepeace B.L. (2018). The Tick Cell Biobank: A global resource for in vitro research on ticks, other arthropods and the pathogens they transmit. Ticks TickBorne Dis..

[158] Monteiro C., Vale F.L., Vieira M.S., Perinotto W.M.D.S., Auad A.M., Dolisnki C., Furlong J., Bittencourt V.R.E.P., Cristina de Azevedo Prata M. (2020). Efficacy of *Heterorhabditis baujardi* (Rhabditida: Heterorhabditidae) against *Rhipicephalus microplus* (Acari: Ixodidae) in presence of susceptible and alternate insect hosts. Biocontrol Sci. Technol..

[159] Samish M., Ginsberg H., Glazer I. (2004). Biological control of ticks. J. Parasitol..

[160] Ben Said M., Galai Y., Canales M., Nijhof A.M., Mhadhbi M., Jedidi M., de la Fuente J., Darghouth M.A. (2012). Hd86, the Bm86 tick protein ortholog in *Hyalomma scupense* (syn. *H. detritum*): Expression in Pichia pastoris and analysis of nucleotides and amino acids sequences variations prior to vaccination trials. Vet. Parasitol..

[161] Galai Y., Canales M., Saïd M.B., Gharbi M., Mhadhbi M., Jedidi M., de la Fuente J., Darghouth M.A. (2012). Efficacy of *Hyalomma scupense* (Hd86) antigen against *Hyalomma excavatum* and *H. scupense* tick infestations in cattle. Vaccine.

[162] Bhowmick B., Han Q. (2020). Understanding tick biology and its implications in anti-tick and transmission blocking vaccines against tick-borne pathogens. Front. Vet. Sci..

[163] Contreras M., Villar M., De La Fuente J. (2019). A vaccinomics approach for the identification of tick protective antigens for the control of *Ixodes ricinus* and *Dermacentor reticulatus* infestations in companion animals. Front. Physiol..

[164] De La Fuente J., Blouin E.F., Manzano-Roman R., Naranjo V., Almazán C., De La Lastra J.M.P., Zivkovic Z., Massung R.F., Jongejan F., Kocan K.M. (2008). Differential expression of the tick protective antigen subolesin in *Anaplasma marginale* and *A. phagocytophilum* infected host cells. Ann. N. Y. Acad. Sci..

[165] de la Fuente J., Ayoubi P., Blouin E.F., Almazán C., Naranjo V., Kocan K.M. (2006). Anaplasmosis: Focusing on host-vector-pathogen interactions for vaccine development. Ann. N. Y. Acad. Sci..

[166] Ojogun N., Kahlon A., Ragland S.A., Troese M.J., Mastronunzio J.E., Walker N.J., VieBrock L., Thomas R.J., Borjesson D.L., Fikrig E. (2012). *Anaplasma phagocytophilum* outer membrane protein A interacts with sialylated glycoproteins to promote infection of mammalian host cells. Infect. Immun..

[167] Kahlon A., Ojogun N., Ragland S.A., Seidman D., Troese M.J., Ottens A.K., Fikring E., Carlyon J.A. (2013). *Anaplasma phagocytophilum* Asp14 is an invasin that interacts with mammalian host cells via its C terminus to facilitate infection. Infect. Immun..

[168] Seidman D., Ojogun N., Walker N.J., Mastronunzio J., Kahlon A., Hebert K.S., Karandashova S., Miller D.P., Tegels B.K., Marconi R.T. (2014). *Anaplasma phagocytophilum* surface protein AipA mediates invasion of mammalian host cells. Cell. Microbiol..

[169] He M., Xu W., Zhang L., Liu Z., Zhu J., Li Y., Wu S., Niu H. (2018). Identification of novel immunoreactive proteins and delineation of a specific epitope of *Anaplasma phagocytophilum*. Microb. Pathog..

[170] Fukui Y., Ohkawa S., Inokuma H. (2018). First molecular detection and phylogenetic analysis of *Anaplasma phagocytophilum* from a clinical case of canine granulocytic anaplasmosis in Japan. Jpn. J. Infect. Dis..

[171] Dantas-Torres F., Otranto D., Marcondes C.B. (2017). Anaplasmosis. Arthropod Borne Disease Switzerland.

[172] Yancey C.B., Diniz P.P.V.P., Breitschwerdt E.B., Hegarty B.C., Wiesen C., Qurollo B.A. (2018). Doxycycline treatment efficacy in dogs with naturally occurring *Anaplasma phagocytophilum* infection. J. Small Anim. Pract..

[173] Hansmann Y., Jaulhac B., Kieffer P., Martinot M., Wurtz E., Dukic R., Argemi X., De Martino S. (2019). Value of PCR, serology, and blood smears for human granulocytic anaplasmosis diagnosis, France. Emerg. Infect. Dis..

